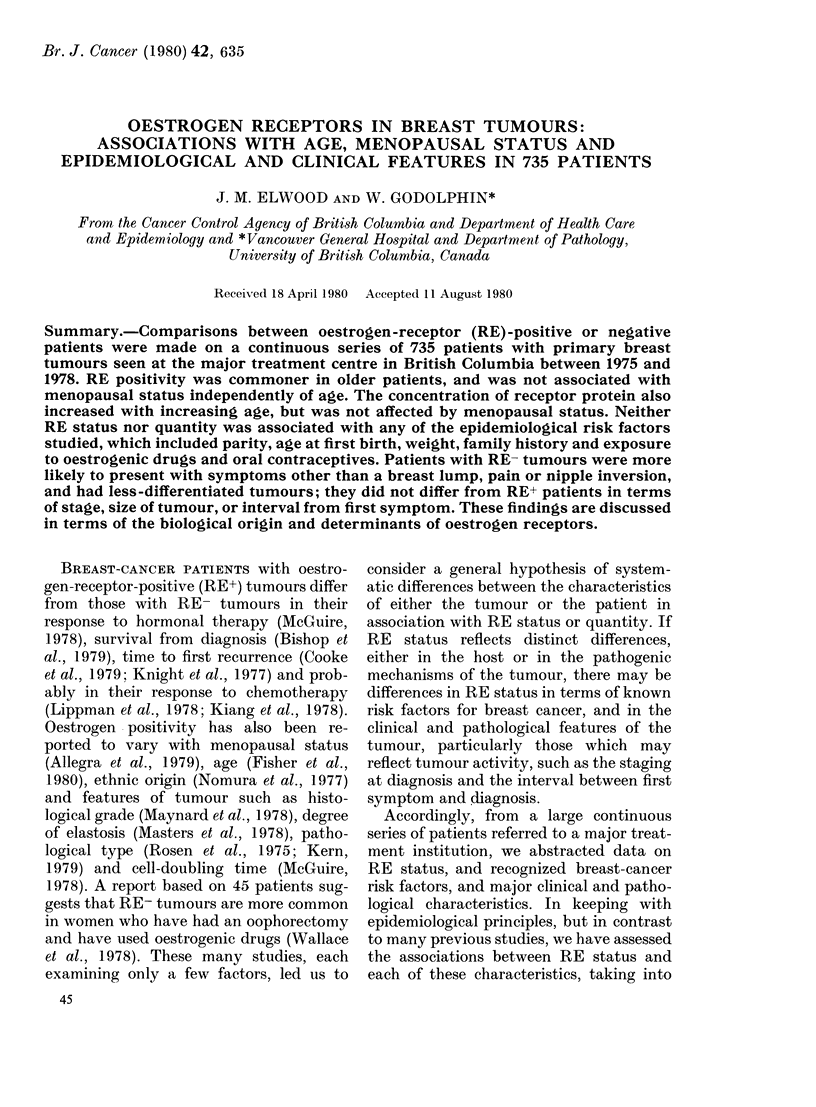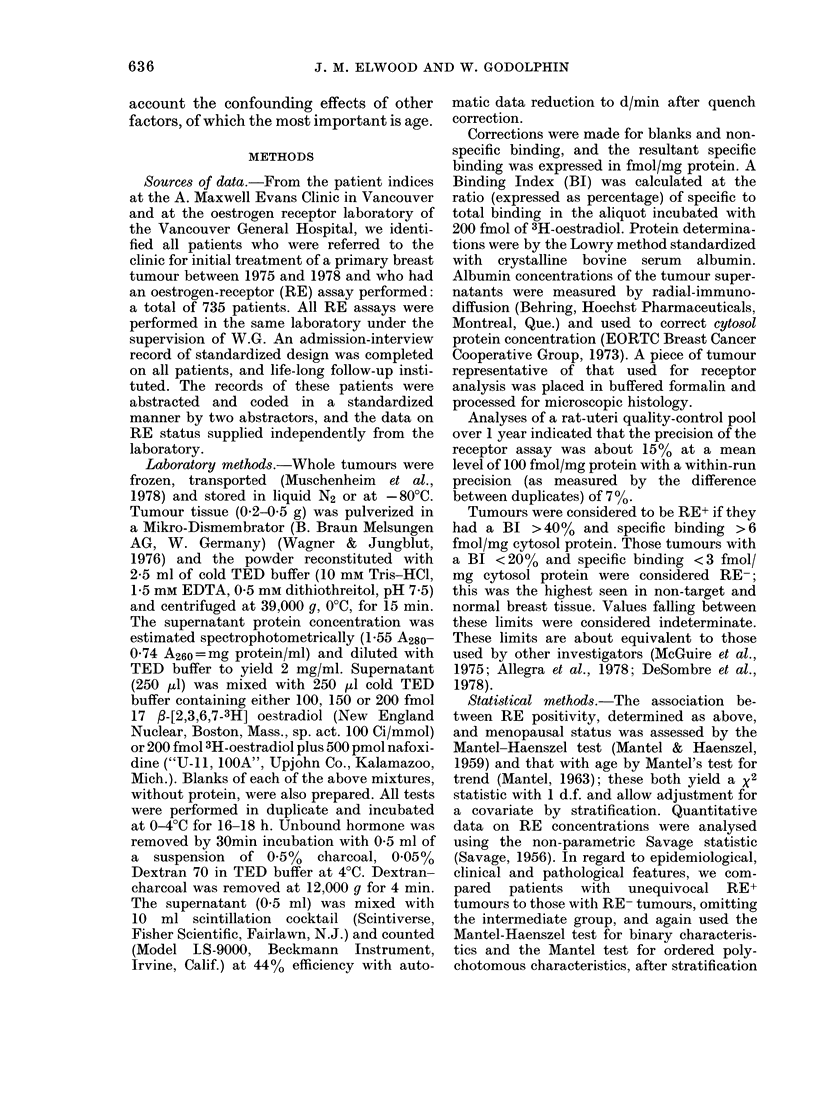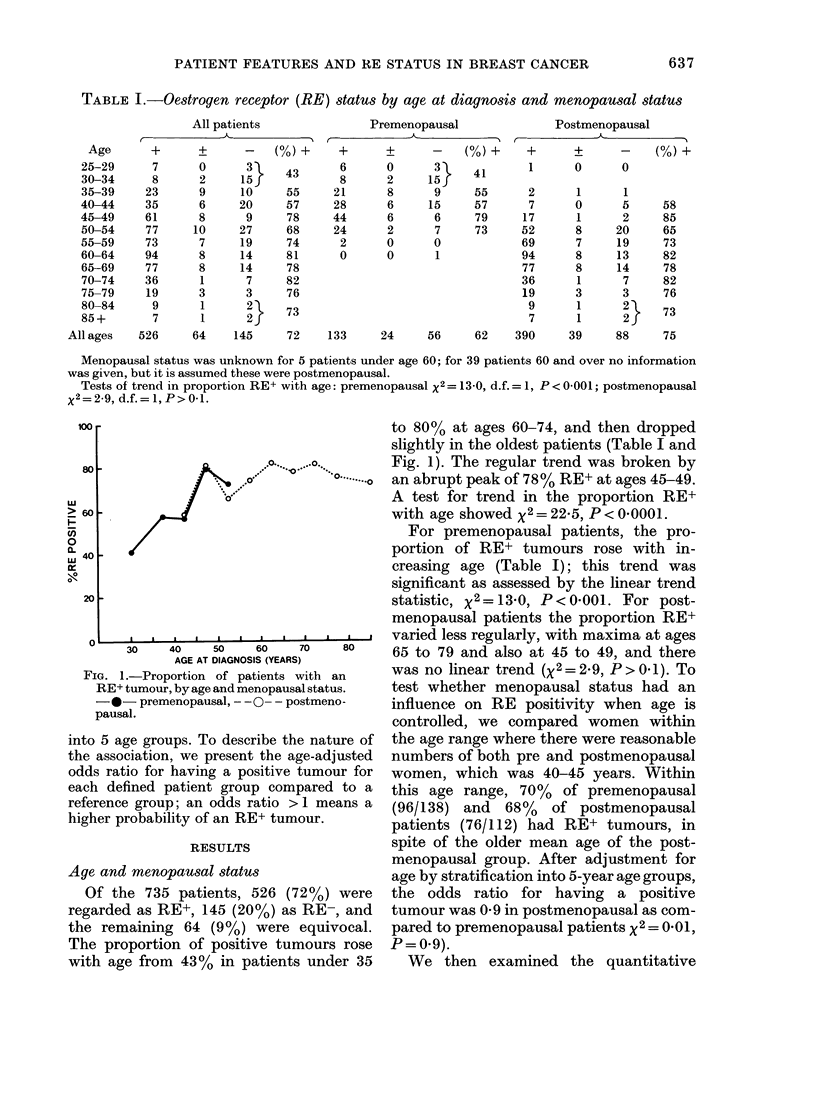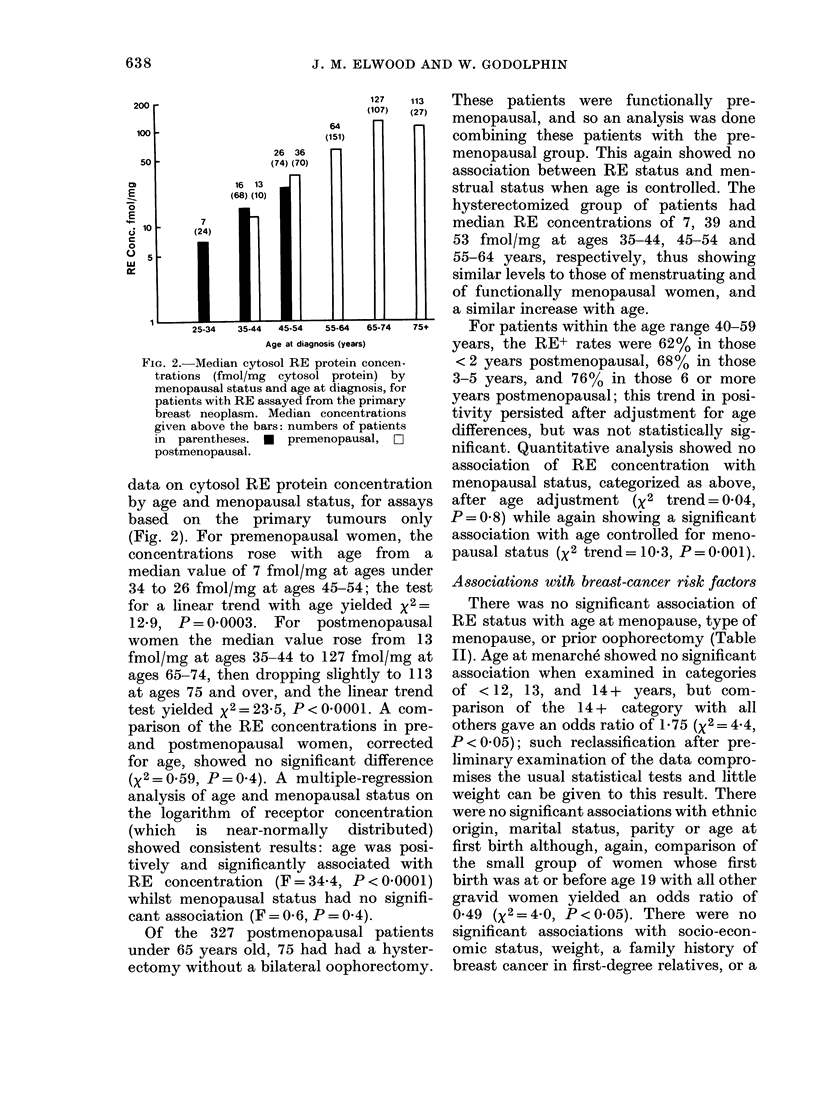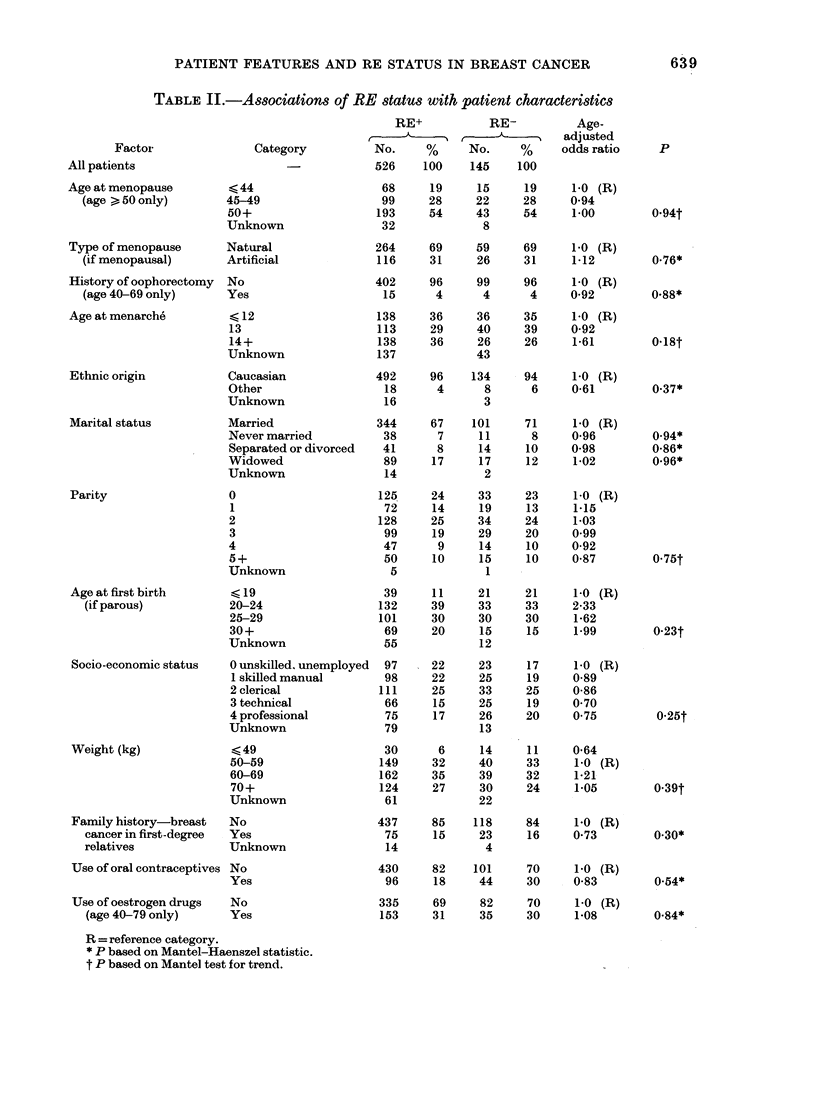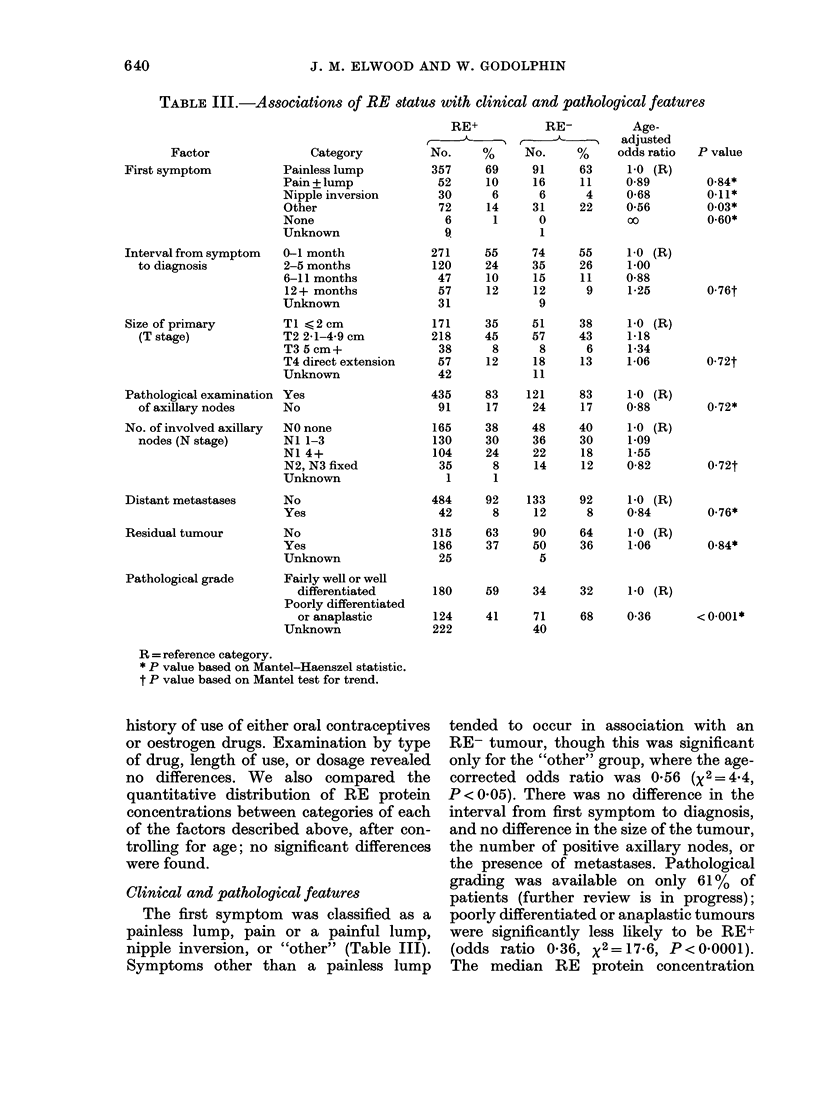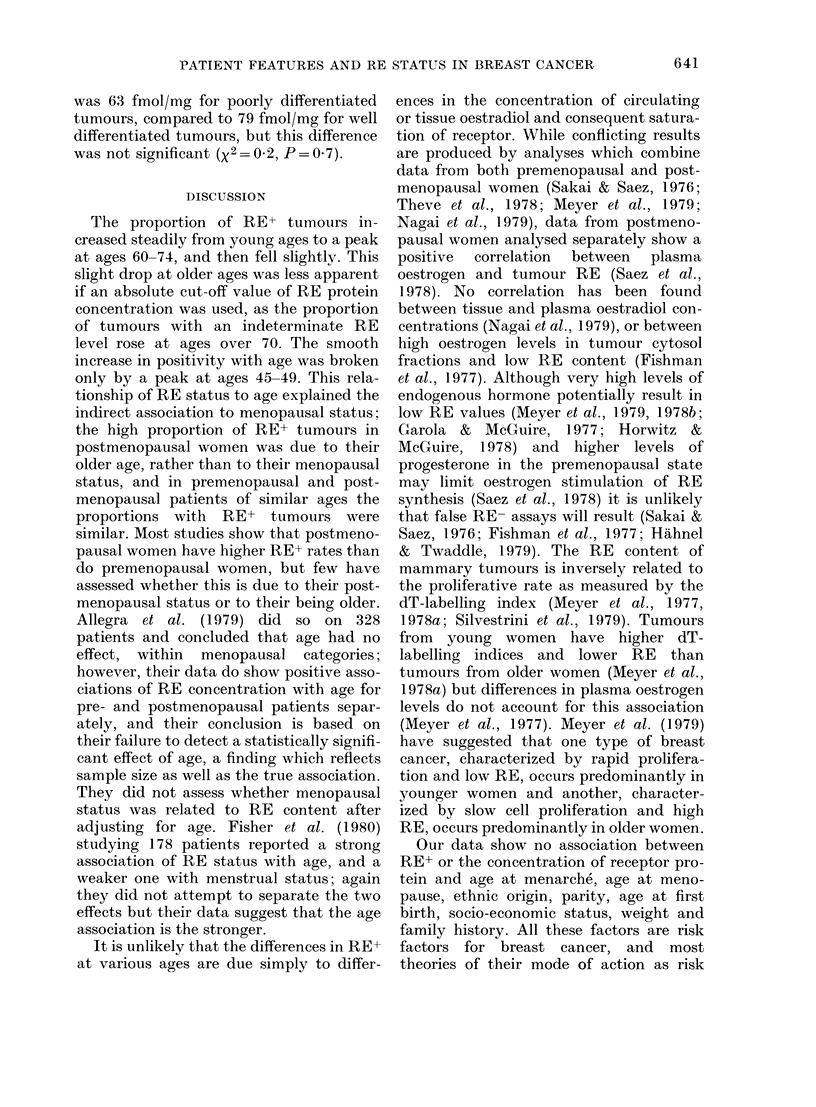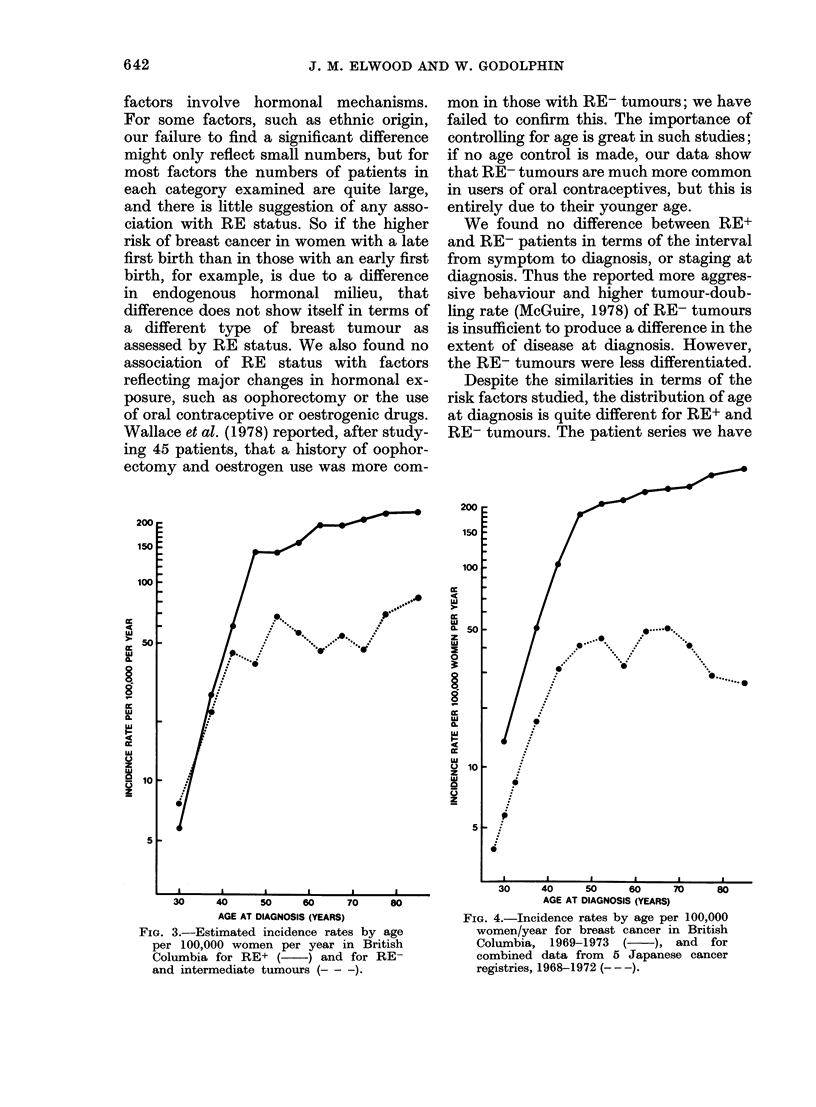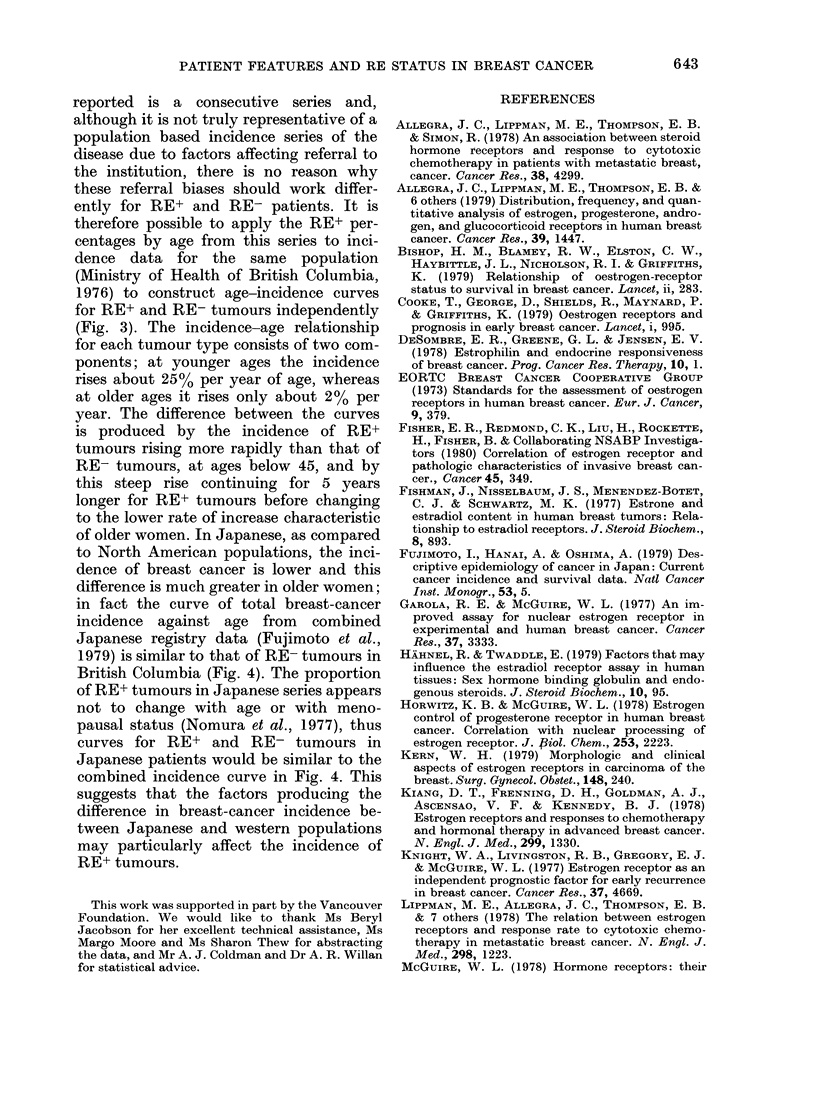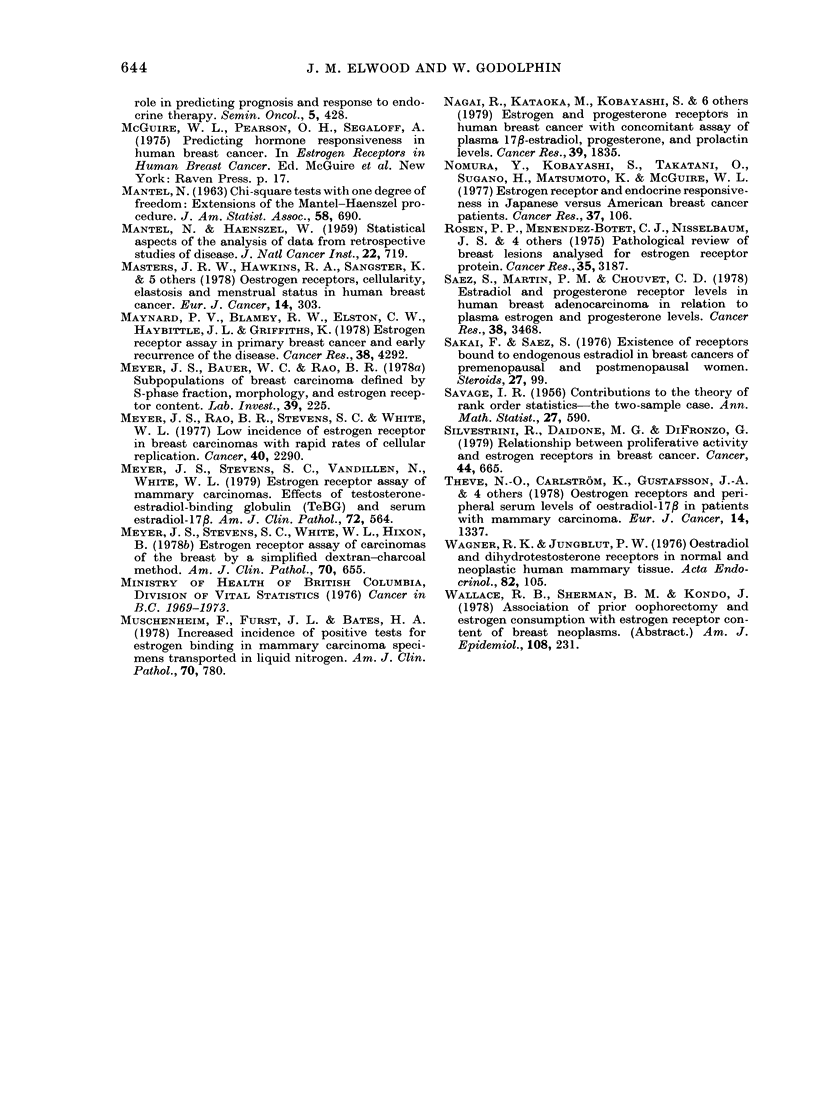# Oestrogen receptors in breast tumours: associations with age, menopausal status and epidemiological and clinical features in 735 patients.

**DOI:** 10.1038/bjc.1980.296

**Published:** 1980-11

**Authors:** J. M. Elwood, W. Godolphin

## Abstract

Comparisons between oestrogen-receptor (RE)-positive or negative patients were made on a continuous series of 735 patients with primary breast tumours seen at the major treatment centre in British Columbia between 1975 and 1978. RE positivity was commoner in older patients, and was not associated with menopausal status independently of age. The concentration of receptor protein also increased with increasing age, but was not affected by menopausal status. Neither RE status nor quantity was associated with any of the epidemiological risk factors studied, which included parity, age at first birth, weight, family history and exposure to oestrogenic drugs and oral contraceptives. Patients with RE- tumours were more likely to present with symptoms other than a breast lump, pain or nipple inversion, and had less-differentiated tumours; they did not differ from RE+ patients in terms of stage, size of tumour, or interval from first symptom. These findings are discussed in terms of the biological origin and determinants of oestrogen receptors.


*Br. J. Cancer* (1980) **42**, 635

### OESTROGEN RECEPTORS IN BREAST TUMOURS: ASSOCIATIONS WITH AGE, MENOPAUSAL STATUS AND EPIDEMIOLOGICAL AND CLINICAL FEATURES IN 735 PATIENTS

J. M. ELWOOD AND W. GODOLPHIN*

*From the Cancer Control Agency of British Columbia and Department of Health Care and Epidemiology and *Vancouver General Hospital and Department of Pathology, University of British Columbia, Canada*

Received 18 April 1980 Accepted 11 August 1980

**Summary.—Comparisons between oestrogen-receptor (RE)-positive or negative patients were made on a continuous series of 735 patients with primary breast tumours seen at the major treatment centre in British Columbia between 1975 and 1978. RE positivity was commoner in older patients, and was not associated with menopausal status independently of age. The concentration of receptor protein also increased with increasing age, but was not affected by menopausal status. Neither RE status nor quantity was associated with any of the epidemiological risk factors studied, which included parity, age at first birth, weight, family history and exposure to oestrogenic drugs and oral contraceptives. Patients with $RE^-$ tumours were more likely to present with symptoms other than a breast lump, pain or nipple inversion, and had less-differentiated tumours; they did not differ from $RE^+$ patients in terms of stage, size of tumour, or interval from first symptom. These findings are discussed in terms of the biological origin and determinants of oestrogen receptors.**

BREAST-CANCER PATIENTS with oestrogen-receptor-positive ($RE^+$) tumours differ from those with $RE^-$ tumours in their response to hormonal therapy (McGuire, 1978), survival from diagnosis (Bishop *et al.*, 1979), time to first recurrence (Cooke *et al.*, 1979; Knight *et al.*, 1977) and probably in their response to chemotherapy (Lippman *et al.*, 1978; Kiang *et al.*, 1978). Oestrogen positivity has also been reported to vary with menopausal status (Allegra *et al.*, 1979), age (Fisher *et al.*, 1980), ethnic origin (Nomura *et al.*, 1977) and features of tumour such as histological grade (Maynard *et al.*, 1978), degree of elastosis (Masters *et al.*, 1978), pathological type (Rosen *et al.*, 1975; Kern, 1979) and cell-doubling time (McGuire, 1978). A report based on 45 patients suggests that $RE^-$ tumours are more common in women who have had an oophorectomy and have used oestrogenic drugs (Wallace *et al.*, 1978). These many studies, each examining only a few factors, led us to consider a general hypothesis of systematic differences between the characteristics of either the tumour or the patient in association with RE status or quantity. If RE status reflects distinct differences, either in the host or in the pathogenic mechanisms of the tumour, there may be differences in RE status in terms of known risk factors for breast cancer, and in the clinical and pathological features of the tumour, particularly those which may reflect tumour activity, such as the staging at diagnosis and the interval between first symptom and diagnosis.

Accordingly, from a large continuous series of patients referred to a major treatment institution, we abstracted data on RE status, and recognized breast-cancer risk factors, and major clinical and pathological characteristics. In keeping with epidemiological principles, but in contrast to many previous studies, we have assessed the associations between RE status and each of these characteristics, taking into account the confounding effects of other factors, of which the most important is age.

### METHODS

*Sources of data.*—From the patient indices at the A. Maxwell Evans Clinic in Vancouver and at the oestrogen receptor laboratory of the Vancouver General Hospital, we identified all patients who were referred to the clinic for initial treatment of a primary breast tumour between 1975 and 1978 and who had an oestrogen-receptor (RE) assay performed: a total of 735 patients. All RE assays were performed in the same laboratory under the supervision of W.G. An admission-interview record of standardized design was completed on all patients, and life-long follow-up instituted. The records of these patients were abstracted and coded in a standardized manner by two abstractors, and the data on RE status supplied independently from the laboratory.

*Laboratory methods.*—Whole tumours were frozen, transported (Muschenheim *et al.*, 1978) and stored in liquid $N_2$ or at −80°C. Tumour tissue (0·2–0·5 g) was pulverized in a Mikro-Dismembrator (B. Braun Melsungen AG, W. Germany) (Wagner & Jungblut, 1976) and the powder reconstituted with 2·5 ml of cold TED buffer (10 mM Tris-HCl, 1·5 mM EDTA, 0·5 mM dithiothreitol, pH 7·5) and centrifuged at 39,000 *g*, 0°C, for 15 min. The supernatant protein concentration was estimated spectrophotometrically (1·55 $A_{280}$–0·74 $A_{260}$ = mg protein/ml) and diluted with TED buffer to yield 2 mg/ml. Supernatant (250 μl) was mixed with 250 μl cold TED buffer containing either 100, 150 or 200 fmol 17 β-[2,3,6,7-$^3$H] oestradiol (New England Nuclear, Boston, Mass., sp. act. 100 Ci/mmol) or 200 fmol $^3$H-oestradiol plus 500 pmol nafoxidine ("U-11, 100A", Upjohn Co., Kalamazoo, Mich.). Blanks of each of the above mixtures, without protein, were also prepared. All tests were performed in duplicate and incubated at 0–4°C for 16–18 h. Unbound hormone was removed by 30min incubation with 0·5 ml of a suspension of 0·5% charcoal, 0·05% Dextran 70 in TED buffer at 4°C. Dextran–charcoal was removed at 12,000 *g* for 4 min. The supernatant (0·5 ml) was mixed with 10 ml scintillation cocktail (Scintiverse, Fisher Scientific, Fairlawn, N.J.) and counted (Model LS-9000, Beckmann Instrument, Irvine, Calif.) at 44% efficiency with automatic data reduction to d/min after quench correction.

Corrections were made for blanks and non-specific binding, and the resultant specific binding was expressed in fmol/mg protein. A Binding Index (BI) was calculated at the ratio (expressed as percentage) of specific to total binding in the aliquot incubated with 200 fmol of $^3$H-oestradiol. Protein determinations were by the Lowry method standardized with crystalline bovine serum albumin. Albumin concentrations of the tumour supernatants were measured by radial-immunodiffusion (Behring, Hoechst Pharmaceuticals, Montreal, Que.) and used to correct *cytosol* protein concentration (EORTC Breast Cancer Cooperative Group, 1973). A piece of tumour representative of that used for receptor analysis was placed in buffered formalin and processed for microscopic histology.

Analyses of a rat-uteri quality-control pool over 1 year indicated that the precision of the receptor assay was about 15% at a mean level of 100 fmol/mg protein with a within-run precision (as measured by the difference between duplicates) of 7%.

Tumours were considered to be $RE^+$ if they had a BI >40% and specific binding >6 fmol/mg cytosol protein. Those tumours with a BI <20% and specific binding <3 fmol/mg cytosol protein were considered $RE^-$; this was the highest seen in non-target and normal breast tissue. Values falling between these limits were considered indeterminate. These limits are about equivalent to those used by other investigators (McGuire *et al.*, 1975; Allegra *et al.*, 1978; DeSombre *et al.*, 1978).

*Statistical methods.*—The association between RE positivity, determined as above, and menopausal status was assessed by the Mantel–Haenszel test (Mantel & Haenszel, 1959) and that with age by Mantel's test for trend (Mantel, 1963); these both yield a $\chi^2$ statistic with 1 d.f. and allow adjustment for a covariate by stratification. Quantitative data on RE concentrations were analysed using the non-parametric Savage statistic (Savage, 1956). In regard to epidemiological, clinical and pathological features, we compared patients with unequivocal $RE^+$ tumours to those with $RE^-$ tumours, omitting the intermediate group, and again used the Mantel-Haenszel test for binary characteristics and the Mantel test for ordered polychotomous characteristics, after stratification

TABLE I.—*Oestrogen receptor (RE) status by age at diagnosis and menopausal status*

| | All patients | | | | Premenopausal | | | | Postmenopausal | | | |
|---|---|---|---|---|---|---|---|---|---|---|---|---|
| Age | + | ± | − | (%) + | + | ± | − | (%) + | + | ± | − | (%) + |
| 25–29 | 7 | 0 | 3 | 43 | 6 | 0 | 3 | 41 | 1 | 0 | 0 | |
| 30–34 | 8 | 2 | 15 | | 8 | 2 | 15 | | | | | |
| 35–39 | 23 | 9 | 10 | 55 | 21 | 8 | 9 | 55 | 2 | 1 | 1 | |
| 40–44 | 35 | 6 | 20 | 57 | 28 | 6 | 15 | 57 | 7 | 0 | 5 | 58 |
| 45–49 | 61 | 8 | 9 | 78 | 44 | 6 | 6 | 79 | 17 | 1 | 2 | 85 |
| 50–54 | 77 | 10 | 27 | 68 | 24 | 2 | 7 | 73 | 52 | 8 | 20 | 65 |
| 55–59 | 73 | 7 | 19 | 74 | 2 | 0 | 0 | | 69 | 7 | 19 | 73 |
| 60–64 | 94 | 8 | 14 | 81 | 0 | 0 | 1 | | 94 | 8 | 13 | 82 |
| 65–69 | 77 | 8 | 14 | 78 | | | | | 77 | 8 | 14 | 78 |
| 70–74 | 36 | 1 | 7 | 82 | | | | | 36 | 1 | 7 | 82 |
| 75–79 | 19 | 3 | 3 | 76 | | | | | 19 | 3 | 3 | 76 |
| 80–84 | 9 | 1 | 2 | 73 | | | | | 9 | 1 | 2 | 73 |
| 85+ | 7 | 1 | 2 | | | | | | 7 | 1 | 2 | |
| All ages | 526 | 64 | 145 | 72 | 133 | 24 | 56 | 62 | 390 | 39 | 88 | 75 |

Menopausal status was unknown for 5 patients under age 60; for 39 patients 60 and over no information was given, but it is assumed these were postmenopausal.

Tests of trend in proportion RE+ with age: premenopausal $\chi^2 = 13{\cdot}0$, d.f. = 1, $P < 0{\cdot}001$; postmenopausal $\chi^2 = 2{\cdot}9$, d.f. = 1, $P > 0{\cdot}1$.

FIG. 1.—Proportion of patients with an RE+ tumour, by age and menopausal status. —●— premenopausal, – –○– – postmenopausal.

into 5 age groups. To describe the nature of the association, we present the age-adjusted odds ratio for having a positive tumour for each defined patient group compared to a reference group; an odds ratio >1 means a higher probability of an RE+ tumour.

### RESULTS

### *Age and menopausal status*

Of the 735 patients, 526 (72%) were regarded as RE+, 145 (20%) as RE−, and the remaining 64 (9%) were equivocal. The proportion of positive tumours rose with age from 43% in patients under 35 to 80% at ages 60–74, and then dropped slightly in the oldest patients (Table I and Fig. 1). The regular trend was broken by an abrupt peak of 78% RE+ at ages 45–49. A test for trend in the proportion RE+ with age showed $\chi^2 = 22{\cdot}5$, $P < 0{\cdot}0001$.

For premenopausal patients, the proportion of RE+ tumours rose with increasing age (Table I); this trend was significant as assessed by the linear trend statistic, $\chi^2 = 13{\cdot}0$, $P < 0{\cdot}001$. For postmenopausal patients the proportion RE+ varied less regularly, with maxima at ages 65 to 79 and also at 45 to 49, and there was no linear trend ($\chi^2 = 2{\cdot}9$, $P > 0{\cdot}1$). To test whether menopausal status had an influence on RE positivity when age is controlled, we compared women within the age range where there were reasonable numbers of both pre and postmenopausal women, which was 40–45 years. Within this age range, 70% of premenopausal (96/138) and 68% of postmenopausal patients (76/112) had RE+ tumours, in spite of the older mean age of the postmenopausal group. After adjustment for age by stratification into 5-year age groups, the odds ratio for having a positive tumour was 0·9 in postmenopausal as compared to premenopausal patients $\chi^2 = 0{\cdot}01$, $P = 0{\cdot}9$).

We then examined the quantitative

FIG. 2.—Median cytosol RE protein concentrations (fmol/mg cytosol protein) by menopausal status and age at diagnosis, for patients with RE assayed from the primary breast neoplasm. Median concentrations given above the bars: numbers of patients in parentheses. ■ premenopausal, □ postmenopausal.

data on cytosol RE protein concentration by age and menopausal status, for assays based on the primary tumours only (Fig. 2). For premenopausal women, the concentrations rose with age from a median value of 7 fmol/mg at ages under 34 to 26 fmol/mg at ages 45–54; the test for a linear trend with age yielded $\chi^2 = 12{\cdot}9$, $P = 0{\cdot}0003$. For postmenopausal women the median value rose from 13 fmol/mg at ages 35–44 to 127 fmol/mg at ages 65–74, then dropping slightly to 113 at ages 75 and over, and the linear trend test yielded $\chi^2 = 23{\cdot}5$, $P < 0{\cdot}0001$. A comparison of the RE concentrations in pre- and postmenopausal women, corrected for age, showed no significant difference ($\chi^2 = 0{\cdot}59$, $P = 0{\cdot}4$). A multiple-regression analysis of age and menopausal status on the logarithm of receptor concentration (which is near-normally distributed) showed consistent results: age was positively and significantly associated with RE concentration ($F = 34{\cdot}4$, $P < 0{\cdot}0001$) whilst menopausal status had no significant association ($F = 0{\cdot}6$, $P = 0{\cdot}4$).

Of the 327 postmenopausal patients under 65 years old, 75 had had a hysterectomy without a bilateral oophorectomy. These patients were functionally premenopausal, and so an analysis was done combining these patients with the premenopausal group. This again showed no association between RE status and menstrual status when age is controlled. The hysterectomized group of patients had median RE concentrations of 7, 39 and 53 fmol/mg at ages 35–44, 45–54 and 55–64 years, respectively, thus showing similar levels to those of menstruating and of functionally menopausal women, and a similar increase with age.

For patients within the age range 40–59 years, the RE$^+$ rates were 62% in those < 2 years postmenopausal, 68% in those 3–5 years, and 76% in those 6 or more years postmenopausal; this trend in positivity persisted after adjustment for age differences, but was not statistically significant. Quantitative analysis showed no association of RE concentration with menopausal status, categorized as above, after age adjustment ($\chi^2$ trend $= 0{\cdot}04$, $P = 0{\cdot}8$) while again showing a significant association with age controlled for menopausal status ($\chi^2$ trend $= 10{\cdot}3$, $P = 0{\cdot}001$).

*Associations with breast-cancer risk factors*

There was no significant association of RE status with age at menopause, type of menopause, or prior oophorectomy (Table II). Age at menarché showed no significant association when examined in categories of < 12, 13, and 14+ years, but comparison of the 14+ category with all others gave an odds ratio of 1·75 ($\chi^2 = 4{\cdot}4$, $P < 0{\cdot}05$); such reclassification after preliminary examination of the data compromises the usual statistical tests and little weight can be given to this result. There were no significant associations with ethnic origin, marital status, parity or age at first birth although, again, comparison of the small group of women whose first birth was at or before age 19 with all other gravid women yielded an odds ratio of 0·49 ($\chi^2 = 4{\cdot}0$, $P < 0{\cdot}05$). There were no significant associations with socio-economic status, weight, a family history of breast cancer in first-degree relatives, or a

TABLE II.—*Associations of RE status with patient characteristics*

| Factor | Category | RE+ No. | RE+ % | RE− No. | RE− % | Age-adjusted odds ratio | P |
|---|---|---|---|---|---|---|---|
| All patients | — | 526 | 100 | 145 | 100 | | |
| Age at menopause (age ⩾50 only) | ⩽44 | 68 | 19 | 15 | 19 | 1·0 (R) | |
| | 45–49 | 99 | 28 | 22 | 28 | 0·94 | |
| | 50+ | 193 | 54 | 43 | 54 | 1·00 | 0·94† |
| | Unknown | 32 | | 8 | | | |
| Type of menopause (if menopausal) | Natural | 264 | 69 | 59 | 69 | 1·0 (R) | |
| | Artificial | 116 | 31 | 26 | 31 | 1·12 | 0·76* |
| History of oophorectomy (age 40–69 only) | No | 402 | 96 | 99 | 96 | 1·0 (R) | |
| | Yes | 15 | 4 | 4 | 4 | 0·92 | 0·88* |
| Age at menarché | ⩽12 | 138 | 36 | 36 | 35 | 1·0 (R) | |
| | 13 | 113 | 29 | 40 | 39 | 0·92 | |
| | 14+ | 138 | 36 | 26 | 26 | 1·61 | 0·18† |
| | Unknown | 137 | | 43 | | | |
| Ethnic origin | Caucasian | 492 | 96 | 134 | 94 | 1·0 (R) | |
| | Other | 18 | 4 | 8 | 6 | 0·61 | 0·37* |
| | Unknown | 16 | | 3 | | | |
| Marital status | Married | 344 | 67 | 101 | 71 | 1·0 (R) | |
| | Never married | 38 | 7 | 11 | 8 | 0·96 | 0·94* |
| | Separated or divorced | 41 | 8 | 14 | 10 | 0·98 | 0·86* |
| | Widowed | 89 | 17 | 17 | 12 | 1·02 | 0·96* |
| | Unknown | 14 | | 2 | | | |
| Parity | 0 | 125 | 24 | 33 | 23 | 1·0 (R) | |
| | 1 | 72 | 14 | 19 | 13 | 1·15 | |
| | 2 | 128 | 25 | 34 | 24 | 1·03 | |
| | 3 | 99 | 19 | 29 | 20 | 0·99 | |
| | 4 | 47 | 9 | 14 | 10 | 0·92 | |
| | 5+ | 50 | 10 | 15 | 10 | 0·87 | 0·75† |
| | Unknown | 5 | | 1 | | | |
| Age at first birth (if parous) | ⩽19 | 39 | 11 | 21 | 21 | 1·0 (R) | |
| | 20–24 | 132 | 39 | 33 | 33 | 2·33 | |
| | 25–29 | 101 | 30 | 30 | 30 | 1·62 | |
| | 30+ | 69 | 20 | 15 | 15 | 1·99 | 0·23† |
| | Unknown | 55 | | 12 | | | |
| Socio-economic status | 0 unskilled, unemployed | 97 | 22 | 23 | 17 | 1·0 (R) | |
| | 1 skilled manual | 98 | 22 | 25 | 19 | 0·89 | |
| | 2 clerical | 111 | 25 | 33 | 25 | 0·86 | |
| | 3 technical | 66 | 15 | 25 | 19 | 0·70 | |
| | 4 professional | 75 | 17 | 26 | 20 | 0·75 | 0·25† |
| | Unknown | 79 | | 13 | | | |
| Weight (kg) | ⩽49 | 30 | 6 | 14 | 11 | 0·64 | |
| | 50–59 | 149 | 32 | 40 | 33 | 1·0 (R) | |
| | 60–69 | 162 | 35 | 39 | 32 | 1·21 | |
| | 70+ | 124 | 27 | 30 | 24 | 1·05 | 0·39† |
| | Unknown | 61 | | 22 | | | |
| Family history—breast cancer in first-degree relatives | No | 437 | 85 | 118 | 84 | 1·0 (R) | |
| | Yes | 75 | 15 | 23 | 16 | 0·73 | 0·30* |
| | Unknown | 14 | | 4 | | | |
| Use of oral contraceptives | No | 430 | 82 | 101 | 70 | 1·0 (R) | |
| | Yes | 96 | 18 | 44 | 30 | 0·83 | 0·54* |
| Use of oestrogen drugs (age 40–79 only) | No | 335 | 69 | 82 | 70 | 1·0 (R) | |
| | Yes | 153 | 31 | 35 | 30 | 1·08 | 0·84* |

R = reference category.
* *P* based on Mantel–Haenszel statistic.
† *P* based on Mantel test for trend.

TABLE III.—*Associations of RE status with clinical and pathological features*

| Factor | Category | RE+ No. | RE+ % | RE− No. | RE− % | Age-adjusted odds ratio | $P$ value |
|---|---|---|---|---|---|---|---|
| First symptom | Painless lump | 357 | 69 | 91 | 63 | 1·0 (R) | |
| | Pain ± lump | 52 | 10 | 16 | 11 | 0·89 | 0·84* |
| | Nipple inversion | 30 | 6 | 6 | 4 | 0·68 | 0·11* |
| | Other | 72 | 14 | 31 | 22 | 0·56 | 0·03* |
| | None | 6 | 1 | 0 | | ∞ | 0·60* |
| | Unknown | 9 | | 1 | | | |
| Interval from symptom to diagnosis | 0–1 month | 271 | 55 | 74 | 55 | 1·0 (R) | |
| | 2–5 months | 120 | 24 | 35 | 26 | 1·00 | |
| | 6–11 months | 47 | 10 | 15 | 11 | 0·88 | |
| | 12+ months | 57 | 12 | 12 | 9 | 1·25 | 0·76† |
| | Unknown | 31 | | 9 | | | |
| Size of primary (T stage) | T1 ⩽2 cm | 171 | 35 | 51 | 38 | 1·0 (R) | |
| | T2 2·1–4·9 cm | 218 | 45 | 57 | 43 | 1·18 | |
| | T3 5 cm+ | 38 | 8 | 8 | 6 | 1·34 | |
| | T4 direct extension | 57 | 12 | 18 | 13 | 1·06 | 0·72† |
| | Unknown | 42 | | 11 | | | |
| Pathological examination of axillary nodes | Yes | 435 | 83 | 121 | 83 | 1·0 (R) | |
| | No | 91 | 17 | 24 | 17 | 0·88 | 0·72* |
| No. of involved axillary nodes (N stage) | N0 none | 165 | 38 | 48 | 40 | 1·0 (R) | |
| | N1 1–3 | 130 | 30 | 36 | 30 | 1·09 | |
| | N1 4+ | 104 | 24 | 22 | 18 | 1·55 | |
| | N2, N3 fixed | 35 | 8 | 14 | 12 | 0·82 | 0·72† |
| | Unknown | 1 | 1 | | | | |
| Distant metastases | No | 484 | 92 | 133 | 92 | 1·0 (R) | |
| | Yes | 42 | 8 | 12 | 8 | 0·84 | 0·76* |
| Residual tumour | No | 315 | 63 | 90 | 64 | 1·0 (R) | |
| | Yes | 186 | 37 | 50 | 36 | 1·06 | 0·84* |
| | Unknown | 25 | | 5 | | | |
| Pathological grade | Fairly well or well differentiated | 180 | 59 | 34 | 32 | 1·0 (R) | |
| | Poorly differentiated or anaplastic | 124 | 41 | 71 | 68 | 0·36 | < 0·001* |
| | Unknown | 222 | | 40 | | | |

R = reference category.
* $P$ value based on Mantel–Haenszel statistic.
† $P$ value based on Mantel test for trend.

history of use of either oral contraceptives or oestrogen drugs. Examination by type of drug, length of use, or dosage revealed no differences. We also compared the quantitative distribution of RE protein concentrations between categories of each of the factors described above, after controlling for age; no significant differences were found.

### *Clinical and pathological features*

The first symptom was classified as a painless lump, pain or a painful lump, nipple inversion, or "other" (Table III). Symptoms other than a painless lump tended to occur in association with an RE− tumour, though this was significant only for the "other" group, where the age-corrected odds ratio was 0·56 ($\chi^2 = 4{\cdot}4$, $P < 0{\cdot}05$). There was no difference in the interval from first symptom to diagnosis, and no difference in the size of the tumour, the number of positive axillary nodes, or the presence of metastases. Pathological grading was available on only 61% of patients (further review is in progress); poorly differentiated or anaplastic tumours were significantly less likely to be RE+ (odds ratio 0·36, $\chi^2 = 17{\cdot}6$, $P < 0{\cdot}0001$). The median RE protein concentration was 63 fmol/mg for poorly differentiated tumours, compared to 79 fmol/mg for well differentiated tumours, but this difference was not significant ($\chi^2 = 0{\cdot}2$, $P = 0{\cdot}7$).

### DISCUSSION

The proportion of $RE^+$ tumours increased steadily from young ages to a peak at ages 60–74, and then fell slightly. This slight drop at older ages was less apparent if an absolute cut-off value of RE protein concentration was used, as the proportion of tumours with an indeterminate RE level rose at ages over 70. The smooth increase in positivity with age was broken only by a peak at ages 45–49. This relationship of RE status to age explained the indirect association to menopausal status; the high proportion of $RE^+$ tumours in postmenopausal women was due to their older age, rather than to their menopausal status, and in premenopausal and postmenopausal patients of similar ages the proportions with $RE^+$ tumours were similar. Most studies show that postmenopausal women have higher $RE^+$ rates than do premenopausal women, but few have assessed whether this is due to their postmenopausal status or to their being older. Allegra *et al.* (1979) did so on 328 patients and concluded that age had no effect, within menopausal categories; however, their data do show positive associations of RE concentration with age for pre- and postmenopausal patients separately, and their conclusion is based on their failure to detect a statistically significant effect of age, a finding which reflects sample size as well as the true association. They did not assess whether menopausal status was related to RE content after adjusting for age. Fisher *et al.* (1980) studying 178 patients reported a strong association of RE status with age, and a weaker one with menstrual status; again they did not attempt to separate the two effects but their data suggest that the age association is the stronger.

It is unlikely that the differences in $RE^+$ at various ages are due simply to differences in the concentration of circulating or tissue oestradiol and consequent saturation of receptor. While conflicting results are produced by analyses which combine data from both premenopausal and postmenopausal women (Sakai & Saez, 1976; Theve *et al.*, 1978; Meyer *et al.*, 1979; Nagai *et al.*, 1979), data from postmenopausal women analysed separately show a positive correlation between plasma oestrogen and tumour RE (Saez *et al.*, 1978). No correlation has been found between tissue and plasma oestradiol concentrations (Nagai *et al.*, 1979), or between high oestrogen levels in tumour cytosol fractions and low RE content (Fishman *et al.*, 1977). Although very high levels of endogenous hormone potentially result in low RE values (Meyer *et al.*, 1979, 1978*b*; Garola & McGuire, 1977; Horwitz & McGuire, 1978) and higher levels of progesterone in the premenopausal state may limit oestrogen stimulation of RE synthesis (Saez *et al.*, 1978) it is unlikely that false $RE^-$ assays will result (Sakai & Saez, 1976; Fishman *et al.*, 1977; Hähnel & Twaddle, 1979). The RE content of mammary tumours is inversely related to the proliferative rate as measured by the dT-labelling index (Meyer *et al.*, 1977, 1978*a*; Silvestrini *et al.*, 1979). Tumours from young women have higher dT-labelling indices and lower RE than tumours from older women (Meyer *et al.*, 1978*a*) but differences in plasma oestrogen levels do not account for this association (Meyer *et al.*, 1977). Meyer *et al.* (1979) have suggested that one type of breast cancer, characterized by rapid proliferation and low RE, occurs predominantly in younger women and another, characterized by slow cell proliferation and high RE, occurs predominantly in older women.

Our data show no association between $RE^+$ or the concentration of receptor protein and age at menarché, age at menopause, ethnic origin, parity, age at first birth, socio-economic status, weight and family history. All these factors are risk factors for breast cancer, and most theories of their mode of action as risk factors involve hormonal mechanisms. For some factors, such as ethnic origin, our failure to find a significant difference might only reflect small numbers, but for most factors the numbers of patients in each category examined are quite large, and there is little suggestion of any association with RE status. So if the higher risk of breast cancer in women with a late first birth than in those with an early first birth, for example, is due to a difference in endogenous hormonal milieu, that difference does not show itself in terms of a different type of breast tumour as assessed by RE status. We also found no association of RE status with factors reflecting major changes in hormonal exposure, such as oophorectomy or the use of oral contraceptive or oestrogenic drugs. Wallace *et al.* (1978) reported, after studying 45 patients, that a history of oophorectomy and oestrogen use was more common in those with $RE^-$ tumours; we have failed to confirm this. The importance of controlling for age is great in such studies; if no age control is made, our data show that $RE^-$ tumours are much more common in users of oral contraceptives, but this is entirely due to their younger age.

We found no difference between $RE^+$ and $RE^-$ patients in terms of the interval from symptom to diagnosis, or staging at diagnosis. Thus the reported more aggressive behaviour and higher tumour-doubling rate (McGuire, 1978) of $RE^-$ tumours is insufficient to produce a difference in the extent of disease at diagnosis. However, the $RE^-$ tumours were less differentiated.

Despite the similarities in terms of the risk factors studied, the distribution of age at diagnosis is quite different for $RE^+$ and $RE^-$ tumours. The patient series we have

FIG. 3.—Estimated incidence rates by age per 100,000 women per year in British Columbia for $RE^+$ (——) and for $RE^-$ and intermediate tumours (- - -).

FIG. 4.—Incidence rates by age per 100,000 women/year for breast cancer in British Columbia, 1969–1973 (——), and for combined data from 5 Japanese cancer registries, 1968–1972 (- - -).

reported is a consecutive series and, although it is not truly representative of a population based incidence series of the disease due to factors affecting referral to the institution, there is no reason why these referral biases should work differently for $RE^+$ and $RE^-$ patients. It is therefore possible to apply the $RE^+$ percentages by age from this series to incidence data for the same population (Ministry of Health of British Columbia, 1976) to construct age–incidence curves for $RE^+$ and $RE^-$ tumours independently (Fig. 3). The incidence–age relationship for each tumour type consists of two components; at younger ages the incidence rises about 25% per year of age, whereas at older ages it rises only about 2% per year. The difference between the curves is produced by the incidence of $RE^+$ tumours rising more rapidly than that of $RE^-$ tumours, at ages below 45, and by this steep rise continuing for 5 years longer for $RE^+$ tumours before changing to the lower rate of increase characteristic of older women. In Japanese, as compared to North American populations, the incidence of breast cancer is lower and this difference is much greater in older women; in fact the curve of total breast-cancer incidence against age from combined Japanese registry data (Fujimoto *et al.*, 1979) is similar to that of $RE^-$ tumours in British Columbia (Fig. 4). The proportion of $RE^+$ tumours in Japanese series appears not to change with age or with menopausal status (Nomura *et al.*, 1977), thus curves for $RE^+$ and $RE^-$ tumours in Japanese patients would be similar to the combined incidence curve in Fig. 4. This suggests that the factors producing the difference in breast-cancer incidence between Japanese and western populations may particularly affect the incidence of $RE^+$ tumours.

This work was supported in part by the Vancouver Foundation. We would like to thank Ms Beryl Jacobson for her excellent technical assistance, Ms Margo Moore and Ms Sharon Thew for abstracting the data, and Mr A. J. Coldman and Dr A. R. Willan for statistical advice.

### REFERENCES

ALLEGRA, J. C., LIPPMAN, M. E., THOMPSON, E. B. & SIMON, R. (1978) An association between steroid hormone receptors and response to cytotoxic chemotherapy in patients with metastatic breast, cancer. *Cancer Res.*, **38**, 4299.

ALLEGRA, J. C., LIPPMAN, M. E., THOMPSON, E. B. & 6 others (1979) Distribution, frequency, and quantitative analysis of estrogen, progesterone, androgen, and glucocorticoid receptors in human breast cancer. *Cancer Res.*, **39**, 1447.

BISHOP, H. M., BLAMEY, R. W., ELSTON, C. W., HAYBITTLE, J. L., NICHOLSON, R. I. & GRIFFITHS, K. (1979) Relationship of oestrogen-receptor status to survival in breast cancer. *Lancet*, ii, 283.

COOKE, T., GEORGE, D., SHIELDS, R., MAYNARD, P. & GRIFFITHS, K. (1979) Oestrogen receptors and prognosis in early breast cancer. *Lancet*, i, 995.

DESOMBRE, E. R., GREENE, G. L. & JENSEN, E. V. (1978) Estrophilin and endocrine responsiveness of breast cancer. *Prog. Cancer Res. Therapy*, **10**, 1.

EORTC BREAST CANCER COOPERATIVE GROUP (1973) Standards for the assessment of oestrogen receptors in human breast cancer. *Eur. J. Cancer*, **9**, 379.

FISHER, E. R., REDMOND, C. K., LIU, H., ROCKETTE, H., FISHER, B. & Collaborating NSABP Investigators (1980) Correlation of estrogen receptor and pathologic characteristics of invasive breast cancer., *Cancer* **45**, 349.

FISHMAN, J., NISSELBAUM, J. S., MENENDEZ-BOTET, C. J. & SCHWARTZ, M. K. (1977) Estrone and estradiol content in human breast tumors: Relationship to estradiol receptors. *J. Steroid Biochem.*, **8**, 893.

FUJIMOTO, I., HANAI, A. & OSHIMA, A. (1979) Descriptive epidemiology of cancer in Japan: Current cancer incidence and survival data. *Natl Cancer Inst. Monogr.*, **53**, 5.

GAROLA, R. E. & MCGUIRE, W. L. (1977) An improved assay for nuclear estrogen receptor in experimental and human breast cancer. *Cancer Res.*, **37**, 3333.

HÄHNEL, R. & TWADDLE, E. (1979) Factors that may influence the estradiol receptor assay in human tissues: Sex hormone binding globulin and endogenous steroids. *J. Steroid Biochem.*, **10**, 95.

HORWITZ, K. B. & MCGUIRE, W. L. (1978) Estrogen control of progesterone receptor in human breast cancer. Correlation with nuclear processing of estrogen receptor. *J. Biol. Chem.*, **253**, 2223.

KERN, W. H. (1979) Morphologic and clinical aspects of estrogen receptors in carcinoma of the breast. *Surg. Gynecol. Obstet.*, **148**, 240.

KIANG, D. T., FRENNING, D. H., GOLDMAN, A. J., ASCENSAO, V. F. & KENNEDY, B. J. (1978) Estrogen receptors and responses to chemotherapy and hormonal therapy in advanced breast cancer. *N. Engl. J. Med.*, **299**, 1330.

KNIGHT, W. A., LIVINGSTON, R. B., GREGORY, E. J. & MCGUIRE, W. L. (1977) Estrogen receptor as an independent prognostic factor for early recurrence in breast cancer. *Cancer Res.*, **37**, 4669.

LIPPMAN, M. E., ALLEGRA, J. C., THOMPSON, E. B. & 7 others (1978) The relation between estrogen receptors and response rate to cytotoxic chemotherapy in metastatic breast cancer. *N. Engl. J. Med.*, **298**, 1223.

MCGUIRE, W. L. (1978) Hormone receptors: their role in predicting prognosis and response to endocrine therapy. *Semin. Oncol.*, **5**, 428.

McGuire, W. L., Pearson, O. H., Segaloff, A. (1975) Predicting hormone responsiveness in human breast cancer. In *Estrogen Receptors in Human Breast Cancer*. Ed. McGuire *et al.* New York: Raven Press. p. 17.

Mantel, N. (1963) Chi-square tests with one degree of freedom: Extensions of the Mantel–Haenszel procedure. *J. Am. Statist. Assoc.*, **58**, 690.

Mantel, N. & Haenszel, W. (1959) Statistical aspects of the analysis of data from retrospective studies of disease. *J. Natl Cancer Inst.*, **22**, 719.

Masters, J. R. W., Hawkins, R. A., Sangster, K. & 5 others (1978) Oestrogen receptors, cellularity, elastosis and menstrual status in human breast cancer. *Eur. J. Cancer*, **14**, 303.

Maynard, P. V., Blamey, R. W., Elston, C. W., Haybittle, J. L. & Griffiths, K. (1978) Estrogen receptor assay in primary breast cancer and early recurrence of the disease. *Cancer Res.*, **38**, 4292.

Meyer, J. S., Bauer, W. C. & Rao, B. R. (1978*a*) Subpopulations of breast carcinoma defined by S-phase fraction, morphology, and estrogen receptor content. *Lab. Invest.*, **39**, 225.

Meyer, J. S., Rao, B. R., Stevens, S. C. & White, W. L. (1977) Low incidence of estrogen receptor in breast carcinomas with rapid rates of cellular replication. *Cancer*, **40**, 2290.

Meyer, J. S., Stevens, S. C., Vandillen, N., White, W. L. (1979) Estrogen receptor assay of mammary carcinomas. Effects of testosterone-estradiol-binding globulin (TeBG) and serum estradiol-17$\beta$. *Am. J. Clin. Pathol.*, **72**, 564.

Meyer, J. S., Stevens, S. C., White, W. L., Hixon, B. (1978*b*) Estrogen receptor assay of carcinomas of the breast by a simplified dextran–charcoal method. *Am. J. Clin. Pathol.*, **70**, 655.

Ministry of Health of British Columbia, Division of Vital Statistics (1976) *Cancer in B.C. 1969–1973.*

Muschenheim, F., Furst, J. L. & Bates, H. A. (1978) Increased incidence of positive tests for estrogen binding in mammary carcinoma specimens transported in liquid nitrogen. *Am. J. Clin. Pathol.*, **70**, 780.

Nagai, R., Kataoka, M., Kobayashi, S. & 6 others (1979) Estrogen and progesterone receptors in human breast cancer with concomitant assay of plasma 17$\beta$-estradiol, progesterone, and prolactin levels. *Cancer Res.*, **39**, 1835.

Nomura, Y., Kobayashi, S., Takatani, O., Sugano, H., Matsumoto, K. & McGuire, W. L. (1977) Estrogen receptor and endocrine responsiveness in Japanese versus American breast cancer patients. *Cancer Res.*, **37**, 106.

Rosen, P. P., Menendez-Botet, C. J., Nisselbaum, J. S. & 4 others (1975) Pathological review of breast lesions analysed for estrogen receptor protein. *Cancer Res.*, **35**, 3187.

Saez, S., Martin, P. M. & Chouvet, C. D. (1978) Estradiol and progesterone receptor levels in human breast adenocarcinoma in relation to plasma estrogen and progesterone levels. *Cancer Res.*, **38**, 3468.

Sakai, F. & Saez, S. (1976) Existence of receptors bound to endogenous estradiol in breast cancers of premenopausal and postmenopausal women. *Steroids*, **27**, 99.

Savage, I. R. (1956) Contributions to the theory of rank order statistics—the two-sample case. *Ann. Math. Statist.*, **27**, 590.

Silvestrini, R., Daidone, M. G. & DiFronzo, G. (1979) Relationship between proliferative activity and estrogen receptors in breast cancer. *Cancer*, **44**, 665.

Theve, N.-O., Carlström, K., Gustafsson, J.-A. & 4 others (1978) Oestrogen receptors and peripheral serum levels of oestradiol-17$\beta$ in patients with mammary carcinoma. *Eur. J. Cancer*, **14**, 1337.

Wagner, R. K. & Jungblut, P. W. (1976) Oestradiol and dihydrotestosterone receptors in normal and neoplastic human mammary tissue. *Acta Endocrinol.*, **82**, 105.

Wallace, R. B., Sherman, B. M. & Kondo, J. (1978) Association of prior oophorectomy and estrogen consumption with estrogen receptor content of breast neoplasms. (Abstract.) *Am. J. Epidemiol.*, **108**, 231.